# Listing Occupational Carcinogens

**DOI:** 10.1289/ehp.113-a152a

**Published:** 2005-03

**Authors:** Alice Freund

**Affiliations:** Mt. Sinai School of Medicine, New York, New York, E-mail: alice.freund@mssm.edu

The review by Siemiatycki et al. (2004) is extremely valuable, and I am sure I will refer to it often in the future. However, I would like clarification on the risk classification of some chemicals. In the text the authors state that some chemicals, such as glass wool, were downgraded in risk between 1987and 2002, from “possible human carcinogen” (group 2B) classification, to unclassifiable (group 3). This contradicts Table 5 (Siemiatycki et al. 2004), where the chemicals are listed as “possible human carcinogens” and the authors cited the 2002 volumes of the *IARC* (International Agency for Research on Cancer) *Monographs*; this gives the impression that this is the most up-to-date classification.
